# Increased Expression of PPAR-γ Modulates Monocytes Into a M2-Like Phenotype in SLE Patients: An Implicative Protective Mechanism and Potential Therapeutic Strategy of Systemic Lupus Erythematosus

**DOI:** 10.3389/fimmu.2020.579372

**Published:** 2021-01-19

**Authors:** Yu Liu, Shuangyan Luo, Yi Zhan, Jiayu Wang, Rui Zhao, Yingjie Li, Jinrong Zeng, Qianjin Lu

**Affiliations:** ^1^ Department of Dermatology, The Second Xiangya Hospital of Central South University, Hunan Key Laboratory of Medical Epigenetics, Changsha, China; ^2^ Xiangya Medical School of Central South University, Changsha, China; ^3^ Department of Dermatology, The Third Xiangya Hospital of Central South University, Changsha, China

**Keywords:** monocytes, systemic lupus erythematosus, PPAR-γ, histone modification, Sirt1

## Abstract

Systemic lupus erythematosus (SLE) is a spectrum of autoimmune disorders characterized by continuous inflammation and the production of autoantibodies. Monocytes, as precursors of dendritic cells and macrophages, are involved in the pathogenesis of SLE, particularly in the inflammatory reactions. Previous studies have proved that Pam3CSK4, as a synthetic ligand of TLR2, could stimulate monocytes to differentiated into a M2-like phenotype which presented immunosuppressive functions. However, the underlying mechanisms remain to be further studied. Here, we reported an increased expression of PPAR-γ in the CD14^+^ monocytes from SLE patients, particularly in the treated group of SLE patients and the group with positive anti-dsDNA antibodies. Additionally, PPAR-γ expression decreased in the SLE patients with skin lesion. Furthermore, we demonstrated that Pam3CSK4 stimulation can decrease the expression of CCR7, CD80, IL-1β, IL-6, IL-12, and NF-κB which were related to the M1-like subset of monocytes and increased the expression of ARG1 which was related to the M2-like subset through upregulated PPAR-γ expression and consequently downregulated NF-κB expression in the CD14^+^ monocytes in a time-dependent manner. ChIP-qPCR results further demonstrated that Pam3CSK4 pretreatment could modulate PPAR-γ expression by regulating histone modification through the inhibition of Sirt1 binding to the PPAR-γ promoter. Taken together, our study indicated a protective role of TLR2/Sirt1/PPAR-γ pathway in the pathogenesis of SLE which provided potential therapeutic strategies.

## Introduction

Systemic lupus erythematosus (SLE) is a complicated autoimmune syndrome that is distinguished by perpetuated inflammation and the loss of self-tolerance leading to the production of autoantibodies produced by autoreactive B cells (1, 2). The disrupted tolerance and impaired immune responses in patients with SLE cause damage to multiple organ systems. Innate immune cells are of significant importance in the initiation and modulation of inflammatory responses and also take part in the regulation of acquired immune system (3). Aberrant function of innate immune cells leads to perpetuated inflammatory reactions, thus leading to organ impairment (4).

Monocytes, as an important component of the innate immune system, can defend the host by differentiating into macrophages and dendritic cells (DCs). Monocytes recruited constantly from the peripheral blood is important for the maintenance of the homeostasis of tissue-resident macrophages and DCs (5). Monocytes express various surface molecules, including cluster of differentiation (CD)14 and CD16. Based on the different expression levels of these two molecules, monocytes are classified into three subsets: the classical subset (CD14^high^CD16^−^), which constitutes the majority of circulating monocytes, the intermediate subset (CD14^+^CD16^+^), and the nonclassical subset (CD14^low^CD16^+^). Among these subsets, the classical subset is characterized by significantly lower levels of toll-like receptors (TLRs) and costimulatory factors but higher levels of CD36 and CD163. Once stimulated, classical monocytes can be rapidly recruited to sites of inflammation and undergo phagocytosis, but they do not support inflammatory responses. The intermediate subset expresses significantly higher levels of TLRs than the other two subsets and is CD80^hi^CD86^hi^ HLA-DR^hi^, and this subset exhibits both phagocytic and proinflammatory properties. The nonclassical subset also expresses significantly higher levels of CD80 and CD86 and is proinflammatory (6, 7). The results of the detection of these monocyte subsets in SLE varies among several studies. There are reports of an expansion of CD14^+^CD16^+^ monocytes or reduced percentages of CD14^low^CD16^+^ monocytes in SLE patients compared to healthy controls, while other researchers reported that there is no significant difference in the percentages of the three subsets of monocytes between SLE patients and healthy individuals (5, 7–9). Nevertheless, it is believed that there are defects in the function of monocytes from SLE patients (10, 11).

In response to microenvironmental stimuli, monocytes can polarize into M1-like or M2-like macrophages. M1-like macrophages are proinflammatory with increased expression of costimulatory factors and production of proinflammatory cytokines, whereas M2-like macrophages are immunosuppressive (12, 13). Recent studies suggest that an imbalance between the M1 and M2 phenotypes is closely related to SLE pathogenesis (14). An increased M1 to M2 ratio is associated with SLE flares and disease activity (12, 15). Adoptive transfer of M2-like macrophages ameliorates SLE-like symptoms in the activated lymphocyte-derived-DNA-induced SLE mouse model (16). Previous research found that the TLR1/TLR2 receptor ligand Pam3CSK4 can skew monocyte differentiation in favor of an immunosuppressive M2 phenotype (17). However, the underlying mechanisms need further exploration.

In this study, we discovered an elevated expression level of PPAR-γ in the circulating monocytes isolated from SLE patients compared to those isolated from healthy controls. Moreover, we confirmed that TLR2 activation by Pam3CSK4 could induce the differentiation of monocytes toward the M2-like phenotype by improving the expression of PPAR-γ. Further study verified that elevated acetylation levels of histone H3 in the PPAR-γ promoter contributed to its increased expression, which was regulated by the decreased enrichment of Sirt1 in the promoter region of PPAR-γ. These findings revealed a protective role of PPAR-γ in SLE patients and the epigenetic regulatory mechanism of the expression of PPAR-γ upon stimuli, which may provide an adjuvant therapeutic target for SLE.

## Methods

### SLE Patients and Healthy Individuals

All the enrolled patients in this study fulfilled at least four of the SLE classification criteria of the American College of Rheumatology (18). Disease activity was assessed by Disease Activity Index (SLEDAI) (19). The SLE patients were recruited from dermatological clinics, and the age- and sex-matched healthy individuals were recruited from staff at the Second Xiangya Hospital. All participants were informed and signed informed consent forms. Peripheral venous blood was collected from all participants. The monocytes were divided by CD14 magnetic beads (CAT 130-050-201) according to the instructions provided by the Miltenyi company; Cellular purity was generally >95% detected with flow cytometer. Demographic data and medical data of all enrolled SLE patients were recorded at the time of blood sample collection (see **Supplementary Table S1**). The Human Ethics Committee of the Second Xiangya Hospital give authority to this study.

### RNA Isolation and qPCR

Monocytes were suspended by Trizol reagent (Thermo Fisher Scientific) to extract total RNAs. Complementary DNAs (cDNAs) were synthesized from 1-μg extracted RNAs using a PrimeScript® RT Reagent Kit with gDNA Eraser (TaKaRa), 2-μl cDNA, 10-μl SYBR Premix Ex TaqTM (TaKaRa), and 400 nM forward and reverse primers formed the reaction mixture of a final volume of 20 μl. Real-time PCR was performed on a LightCycler® 96 System (Roche). The relative gene expression was normalized to GAPDH through the comparative CT method. All the primer sequences used in our study are provided in **Supplementary Table S2**.

### Western Blotting

At least 3 × 10^6^ monocytes were lysed in radio immunoprecipitation assay (RIPA) buffer containing a proteinase and phosphatase inhibitor (Beyotime) for 30 min at 4°C. Lysates were centrifuged for 15 min at 14,000g at 4°C and the supernatant was discarded. Protein concentration in cell lysates were measured via the Bradford assay (HyClone-Pierce, USA). Proteins were separated by electrophoresis using 12% vertical dodecyl sulfate-polyacrylamide gel and transferred onto nitrocellulose membranes (Millipore, USA). The PVDF membranes was immersed in 5% skim milk for 1 h at room temperature and then immunoblotted with primary antibodies, icluding rabbit anti-human P50 (ab7971, 1:5,000, Abcam, USA) or mouse anti-human PPAR-γ antibody (ab41928, 1:1,000, Abcam, USA) for 12–16 h at 4°C, followed by incubation with the secondary Goat anti-mouse or anti-rabbit IgG antibody (H&L) (GenScript, USA). The band intensity was measured by an ECL Western blot detection kit (Thermo Scientific, USA). The expression level of PPAR-γ and P50 was quantified by densitometry with normalization to GAPDH.

### Flow Cytometry

Surface markers and cytokines were detected using an FACS Canto II cell analyzer (BD Biosciences, San Jose, California). CD14 on monocytes was labeled with anti- APC anti-human CD14 (BD Biosciences, Cat. No. 325607). For cytokines detection, supernatant of CD14^+^ cells stimulated by Pam3CSK4 for 5 days were collected. The concentrations of proinflammatory cytokines secreted by CD14^+^ cells were detected by a Legendplex Kit (human inflammation panel, Cat. No. 740118, Biolegend) according to the manufacturer’s instructions. In brief, the supernatant was incubated with buffer, antibodies-conjugated microbeads and biotinylated detection antibodies for 2 h. Then, we added PE-labeled streptavidin (SA-PE) into the mixture to incubate for 30 min. After washed, the samples were detected by the FACS Canto II cell analyzer. The results were collected and analyzed with FlowJo software (FlowJo LLC, Ashland, Oregon).

### Chromatin Immunoprecipitation Assays

ChIP analysis was conducted with a ChIP Assay Kit (Millipore) following the manufacturer’s instructions. Sorted monocytes were cross-linked in 1% formaldehyde for 10 min at room temperature, and then, we added glycine to stop the reaction. The fixed cells were washed with 20 ml ice-cold phosphate-buffered saline twice and then lysed. The cell lysates were centrifuged and resuspended before being sonicated to generate 500- to 1,000-bp fragments. After that, the indicated antibodies were added and incubated with the sheared DNA. The immune compounds were precipitated with protein A agarose beads, and then washed and eluted. The precipitated DNA was purified, and then, the target DNA was amplified by qPCR. The primers used are presented in **Supplementary Table S3**. Anti-PPAR-γ (Abcam, ab41928) and anti-SIRT1 antibodies (Abcam, ab12193) were of ChIP grade and were purchased from Abcam. An anti-histone H3ac (pan-acetyl) antibody (pAb) (cat. no. 39139) and an anti-histone H4ac (pan-acetyl) antibody (pAb) (cat. no. 39243) were purchased from Active Motif.

### Pam3CSK4 Stimulation and Inhibitor Treatment

Isolated human monocytes were equally divided into experimental and control groups. A total of 3 × 10^6^ monocytes in each group were cultured in six-well plates and activated by 5 μg/ml Pam3CSK4 (Invivogen, CAS No. 112208-00-1) or a negative control. The cells were harvested for mRNA and protein analysis 5 days later. The supernatant was collected to detect the levels of secreted inflammatory cytokines. For the inhibitor treatment experiment, the monocytes in the experimental group were pretreated with the PPAR-γ inhibitor T0070709 (10 μmol) for 2 h, and then, Pam3CSK4 (5 μg/ml) was added to stimulate the monocytes in both groups. After 5 days, the cells were harvested for mRNA and protein analysis.

### Statistical Analysis

The data are presented as mean± SEM. The Student’s *t* test or Kolmogorov-Smirnov test was conducted to compare the continuous normally distributed variables between two groups based on the sample size, and the one-way ANOVA was conducted to compare the variables among multiple groups. The paired Student’s t-test was conducted to compare variables between different culture conditions in *in vitro* assays. Mann-Whitney U test was conducted to compare the variables that were not normally distributed. Pearson’s correlation was used to analyze linear relationship between two variables. All of the diagrams and graphs illustrating cumulative data were created with GraphPad Prism version 6.0 software. Statistical significance is presented as *P < 0.05, **P < 0.01 and ***P < 0.001.

## Results

### PPAR-γ Expression Is Increased in CD14^+^ Monocytes From SLE Patients

**Figure 1:**
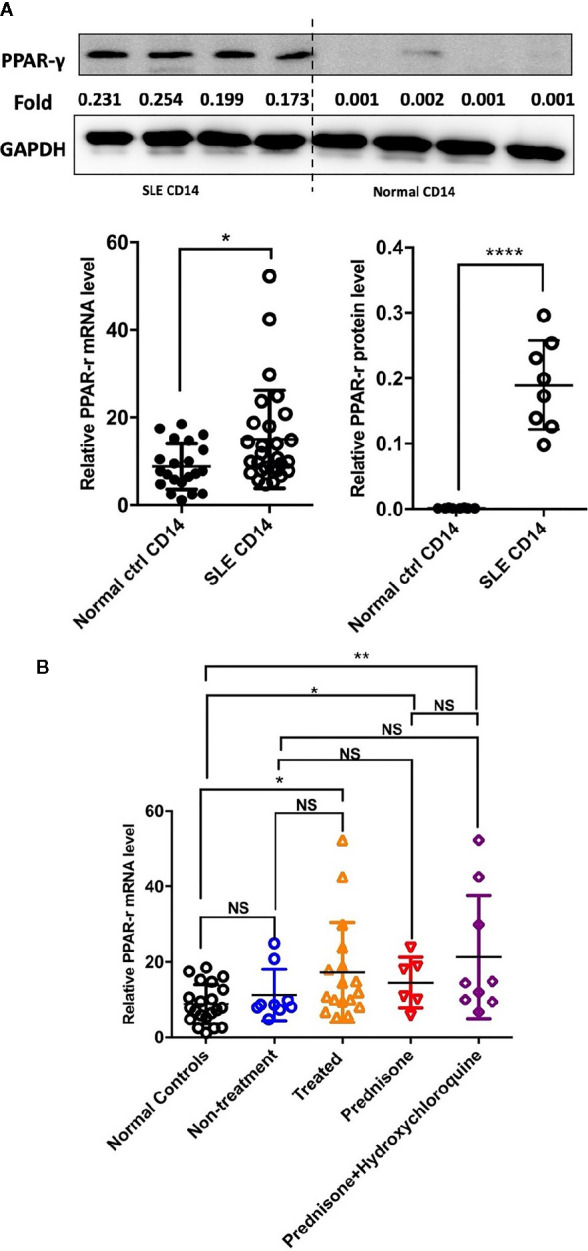
PPAR-γ expression is increased in the CD14^+^ monocytes from SLE patients. **(A)** Both the mRNA level (n1 = 21, n2 = 28) and the protein level (n1, n2 = 8) of PPAR-γ were significantly higher in the circulating CD14^+^ monocytes derived from SLE patients than healthy controls. One representative blot is shown. **(B)** The mRNA expression level of PPAR-γ was significantly increased in the CD14^+^ monocytes from the treated SLE patients (n = 17) compared with those from the normal controls (n = 21), and glucocorticoid (n = 6) or combined with hydroxychloroquine (n = 9) could elevate the expression of PPAR-γ in the SLE patients. RT-PCR results were normalized to GAPDH. Each symbol represents an individual healthy control or patient (n1 represents healthy controls, n2 represents SLE patients. *P < 0.05, **P < 0.01, ****P < 0.0001).

**Figure 2:**
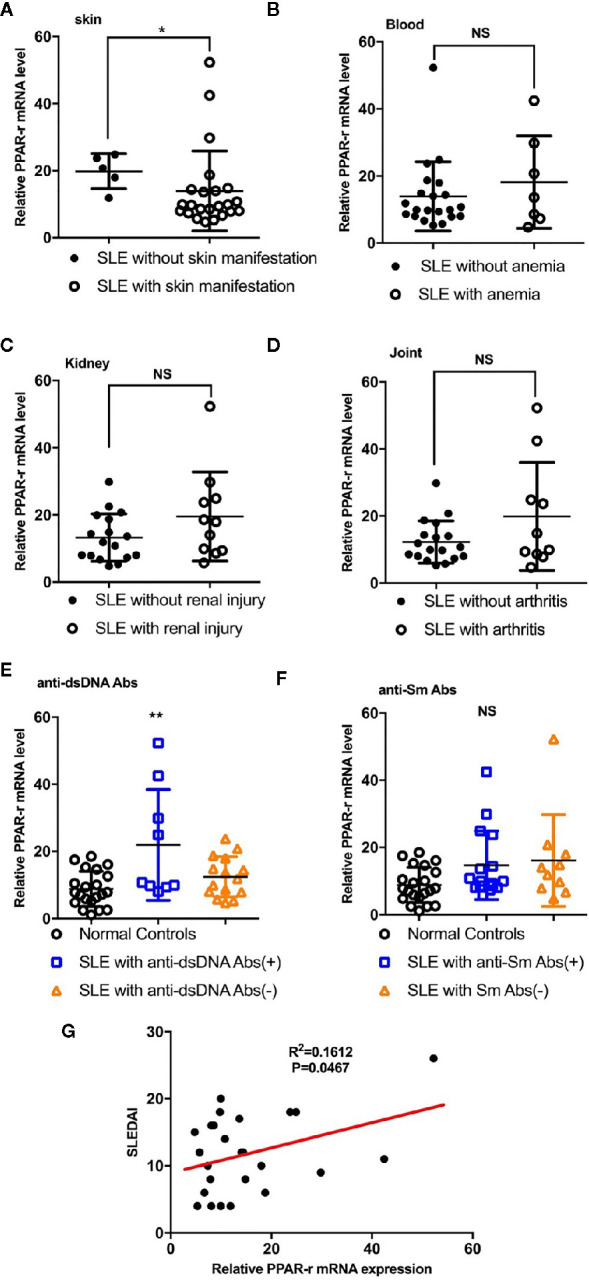
Correlations between PPAR-γ levels and phenotypes, autoantibody levels and SLEDAI. **(A)** PPAR-γ expression in the SLE patients without skin manifestations was higher than that in the SLE patients with skin manifestations (n = 23). **(B**–**D)** PPAR-γ expression in the SLE patients with involvement of the blood system **(B)** (n = 7), kidney **(C)** (n **=** 11), and arthrosis **(D)** (n **=** 10) showed no significant differences compared with that in their control groups. **(E)** PPAR-γ expression in the anti-dsDNA Abs-positive SLE (n = 9) patients was higher than that in the anti-dsDNA Abs-negative SLE patients (n = 15). **(F)** The expression of PPAR-γ in the SLE patients was not affected by anti-Sm Abs (n = 16 for anti-Sm Abs positive patients and n = 10 for anti-Sm Abs negative patients). **(G)** The correlation between PPAR-γ expression and SLEDAI (n = 25). The RT-PCR results were normalized to GAPDH. Each symbol represents an individual healthy control or patient (ns: no significance, *p < 0.05, **p < 0.01).

Detection of the mRNA level of PPAR-γ in the circulating CD14^+^ monocytes demonstrated that PPAR-γ expression is upregulated in SLE patients when compared with healthy controls (**Figure 1A**). To further understand the effects of various medications on the expression of PPAR-γ, we analyzed the mRNA expression level of PPAR-γ in the CD14^+^ monocytes from SLE patients treated with different drugs, including 6 patients treated with glucocorticoids, 9 patients treated with glucocorticoids combined with hydroxychloroquine, and 9 untreated patients; CD14^+^ monocytes from 21 healthy controls were also included in this experiment. Notably, the mRNA expression levels of PPAR-γ between the normal controls and the untreated SLE patients showed no statistical significance. However, the PPAR-γ expression in the CD14^+^ monocytes increased significantly in the treated group compared with the control group, and glucocorticoid or hydroxychloroquine treatment elevated the expression level of PPAR-γ in CD14^+^ monocytes (**Figure 1B**). Furthermore, we analyzed the mRNA level of PPAR-γ in CD14^+^ monocytes from SLE patients of different phenotypes. The results showed that PPAR-γ expression decreased in the SLE patients with involvement of skin (**Figure 2A**). However, the mRNA level of PPAR-γ showed no statistical difference between the SLE patients with or without involvement of the blood system, kidney, and joints (**Figures 2B–D**). In addition, we further analyzed the association between autoantibodies and the expression of PPAR-γ in SLE circulating CD14^+^ monocytes and found a higher mRNA level of PPAR-γ in the SLE patients with positive anti-dsDNA Abs compared to those with negative anti-dsDNA Abs (**Figure 2E**). However, there was no significant difference of the PPAR-γ expression in CD14^+^ monocytes between the groups of SLE patients with or without anti-Sm Abs (**Figure 2F**). Moreover, there lacked a strong association between PPAR-γ expression and the SLE disease activity index (SLEDAI) as the R squared was only 0.1612 (**Figure 2G**).

### TLR2 Stimulation Drives Monocytes to Differentiate Into an M2-Like Phenotype

**Figure 3:**
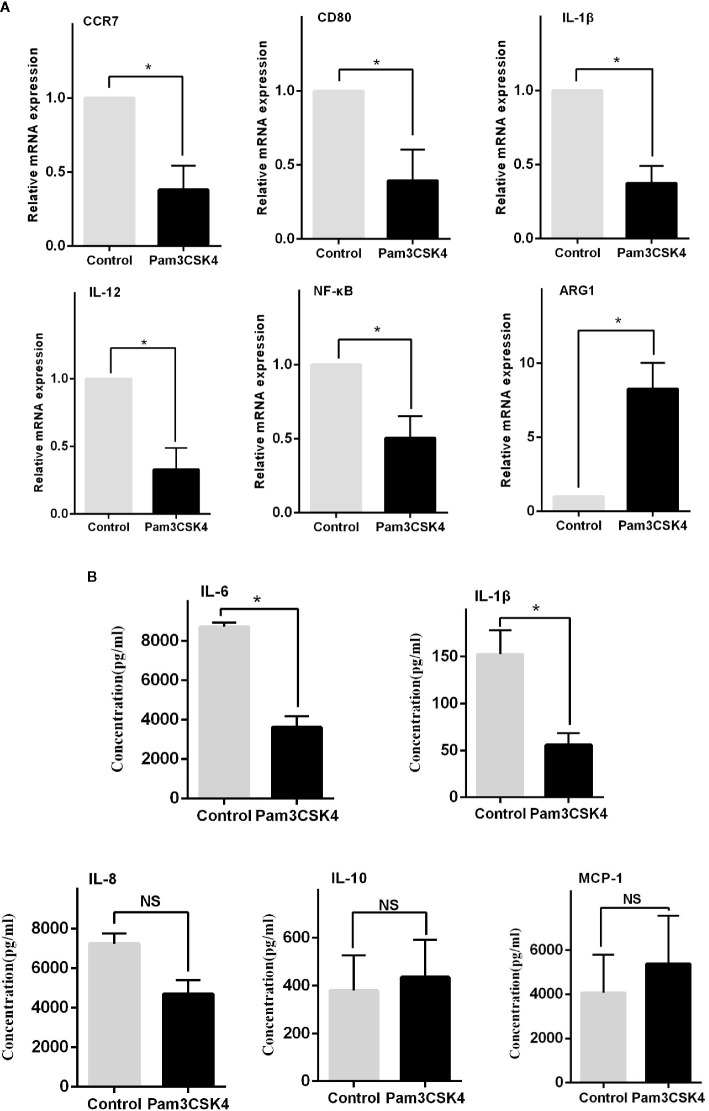
TLR2 stimulation by Pam3CSK4 drives monocytes to differentiate into the M2-like phenotype. **(A)** The RT-PCR results showed that the expression levels of critical proinflammatory cytokines, including CCR7, CD80, IL-1β, IL-12, and NF-κB, significantly decreased and that the expression level of ARG1 increased in the Pam3CSK4-treated group compared with the control group. **(B)** Flow cytometry analysis revealed inhibition of the secretion of IL-1β and IL-6 after Pam3CSK4 treatment while the secreted levels of IL-8, IL-10, and MCP-1 were not decreased. RT-PCR results were normalized to GAPDH. Data represent the mean of three independent experiments (*P < 0.05, NS: No statistical significance and *P* > 0.05).

In addition to the abnormal activity of T and B cells, the imbalance of macrophage subsets is emerging to be an important player in the development of SLE (20). Therefore, inducing monocytes to preferentially shift from an inflammatory M1-like phenotype into an immunosuppressive M2-like phenotype may be a potential therapeutic strategy. Recent work suggested that Pam3CSK4 could normalize the M1:M2 ratio in SLE patients through activating TLR2/1 (17). However, the exact regulatory mechanism is unclear. In our study, we sorted and cultured CD14^+^ monocytes from healthy controls, and we stimulated these cells *in vitro* with phosphate buffered saline (PBS) and Pam3CSK4 for 5 days. We then detected the mRNA expression levels of critical proinflammatory cytokines by Real-time Polymerase Chain Reaction (RT-PCR) including C-C chemokine receptor (CCR)7, Arginase-1 (Arg-1), CD80, Interleukin (IL)-1β, IL-12, and nuclear transcription factor-κB (NF-κB). The results showed that the Pam3CSK4-treated group significantly suppressed the transcription of CCR7, CD80, IL-1β, IL-12, and NF-κB, and induced the Arg-1 expression (**Figure 3A**). In addition, we collected the cell culture supernatants to analyze the secreted levels of proinflammatory cytokines by flow cytometry and found similar inhibition of the secretion of IL-1β and IL-6 (**Figure 3B**). Taken together, these results showed that the molecules related to the M1-like subset were decreased, and the molecules related to the M2-like subset were increased. These results suggested that Pam3CSK4 pretreatment drove monocytes to differentiate toward the M2-like phenotype.

### Pam3SCK4 Induces Monocyte Polarization Accompanied by Increased PPAR-γ Expression

**Figure 4:**
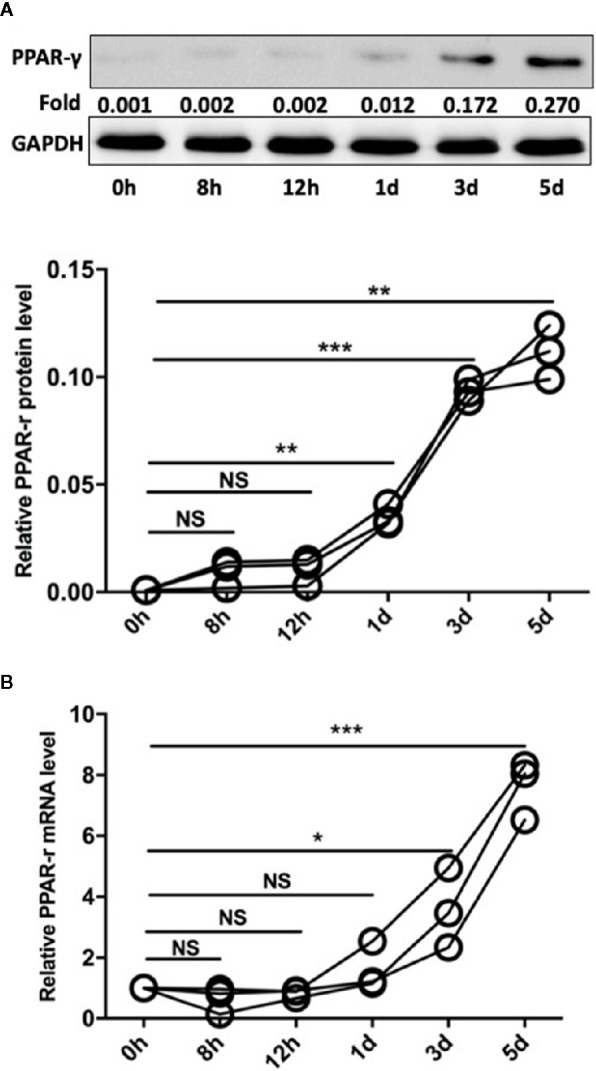
Pam3CSK4 induces monocyte polarization accompanied by increased PPAR-γ expression. **(A)** The Western blot and **(B)** RT-PCR results showed that Pam3CSK4 stimulation elevated PPAR-γ expression in the CD14^+^ monocytes from SLE patients in a time-dependent manner. RT-PCR and Western blot results were normalized to GAPDH. Data represent the mean of three independent experiments (*P < 0.05, **P < 0.01, ***P < 0.001, NS: No statistical significance and *P* > 0.05).

PPAR-γ is a transcription factor (TF) with immunomodulatory properties and has been proposed to promote the expansion of the M2 subpopulation (21). In our study, monocytes were isolated from the peripheral blood mononuclear cells (PBMCs) of SLE patients by CD14 magnetic beads. Then, Pam3CSK4 (5 μg/ml) was utilized to stimulate the TLR2 receptors on monocytes, and the cells were harvested after 8 h, 12 h, 1 day, 3 days, and 5 days. We measured the mRNA and protein levels of PPAR-γ by qPCR and Western blot, respectively. The results showed that Pam3CSK4 pretreatment elevated PPAR-γ expression in the CD14^+^ monocytes from SLE patients in a time-dependent manner (**Figures 4A, B**).

### PPAR-γ Inhibitor Prevents Monocyte Polarization Toward the M2-Like Phenotype

**Figure 5:**
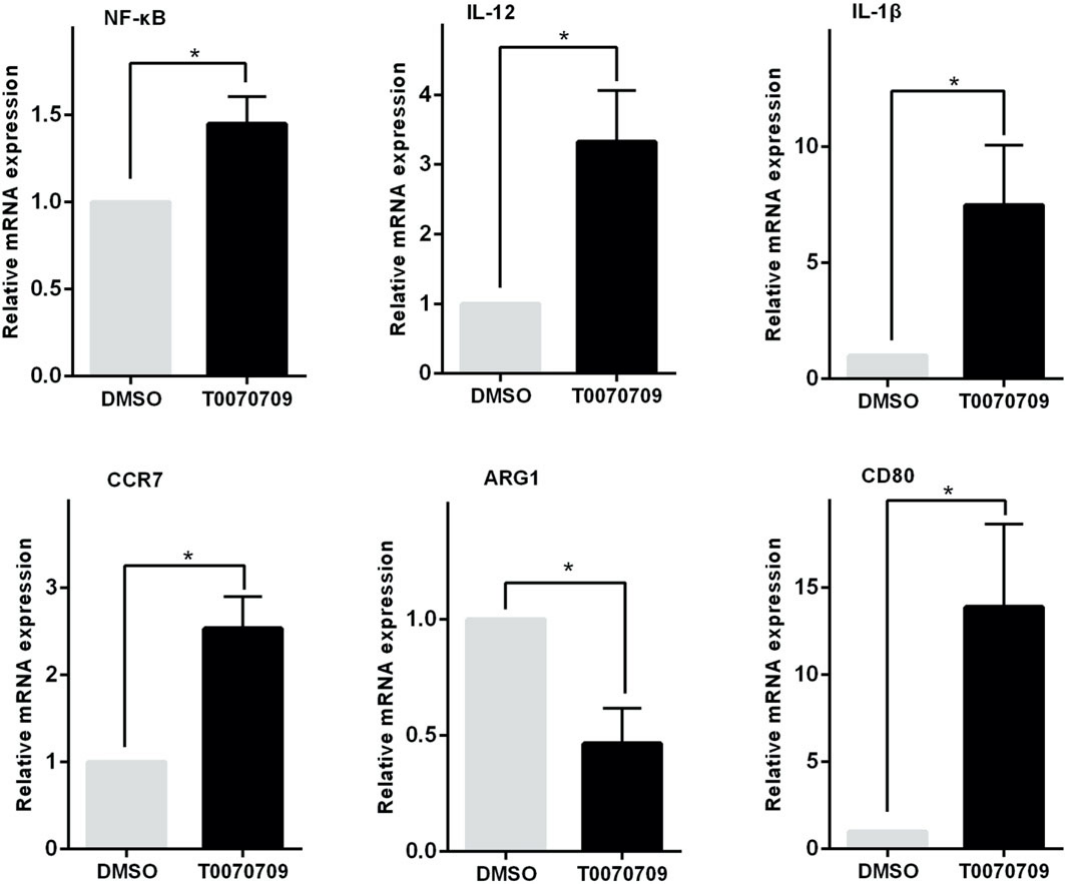
PPAR-γ inhibition prevents monocytes from polarizing toward the M2-like phenotype. The transcription levels of the M1 or M2-like phenotype-associated cytokines were detected by RT-PCR *in vitro*. The expression levels of CCR7, CD80, IL-1β, IL-12, and NF-κB were increased while the expression level of ARG1 was decreased in the PPAR-γ inhibitor T0070709 (10 μmol)-pretreated group compared with the control group. RT-PCR results were normalized to GAPDH. Data represent the mean of three independent experiments (*P < 0.05).

Similarly, when the monocytes were pretreated with the PPAR-γ inhibitor T0070709 (10 μmol) for 2 h, Pam3CSK4 failed to induce monocyte polarization toward the M2-like phenotype. The transcription levels of CCR7, CD80, IL-1β, IL-12, and NF-κB were promoted, while the level of Arg-1 was decreased (**Figure 5**).

### Pam3SCK4 Stimulation Drives Monocytes Polarization Toward the M2-Like Phenotype by the PPAR-γ/NF-κB Pathway

**Figure 6:**
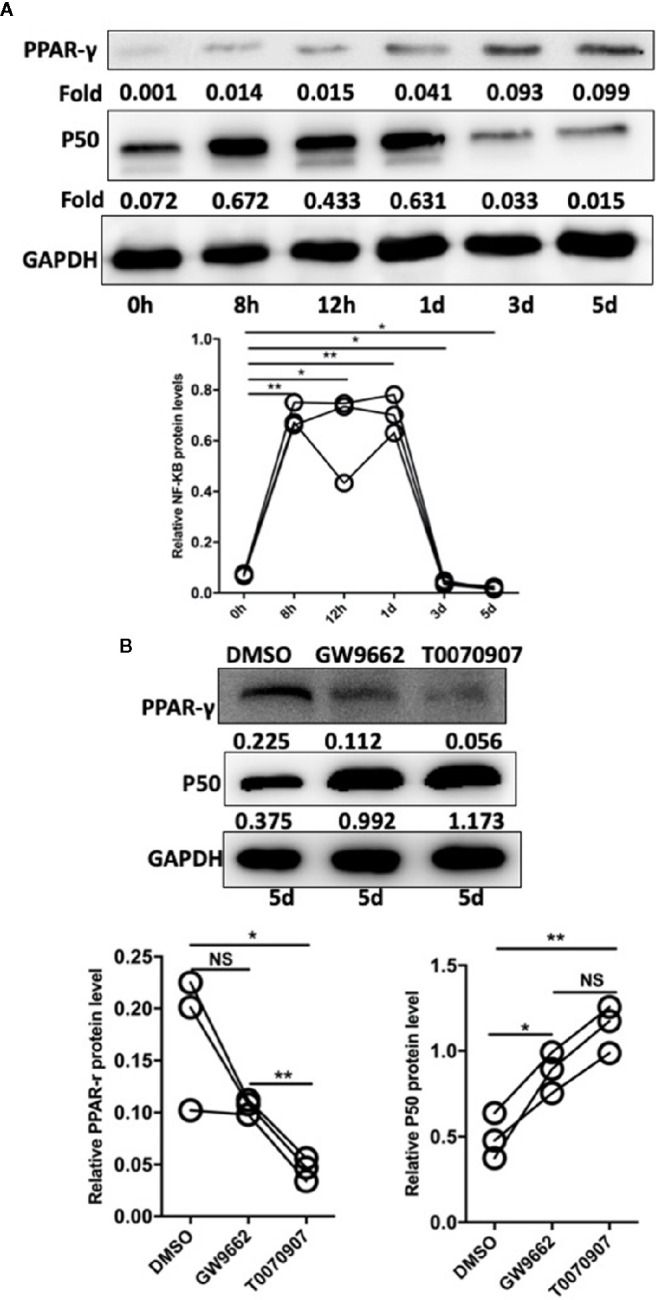
Pam3SCK4 stimulation drives monocytes to differentiate toward the M2-like phenotype via the PPAR-γ/NF-κB pathway. **(A)** The results of western blot showed that the expression level of NF-κB was rapidly upregulated within 8 h after the Pam3SCK4 stimulation, while it rapidly decreased 3 days later. Meanwhile the expression level of PPAR-γ increased gradually. **(B)** PPAR-γ inhibitors T0070709 and GW9662 could inhibit the protein level of PPAR-γ while increase the expression of NF-κB on the fifth day. The western blot results were normalized to GAPDH. Data represent the mean of three independent experiments. (*P < 0.05, **P < 0.01). NS, No statistical significance.

In studies of DCs, the NF-κB pathway is indicated to be involved in the regulation of TLR- and PPAR-γ-modulated signaling (22). Based on the cell experiments described above, after the sorted monocytes were treated with Pam3CSK4, we also detected the expression level of NF-κB. The results showed that the expression level of NF-κB was rapidly initially upregulated by Pam3SCK4 stimulation but then promptly decreased after 3 days, which was possibly due to the activation of PPAR-γ (**Figure 6A**). To confirm this conclusion, we designed another *in vitro* experiment. We pretreated the monocytes with the PPAR-γ inhibitors T0070709 and GW9662 before adding Pam3CSK4. To detect the expression levels of PPAR-γ and NF-κB, we performed Western blotting 5 days later. As expected, the inhibition of PPAR-γ reversed the Pam3SCK4-induced decreased expression of NF-κB (**Figure 6B**).

### Histone Deacetylase Sirt1 Modulates PPAR-γ Expression by Increasing H3 Acetylation

**Figure 7:**
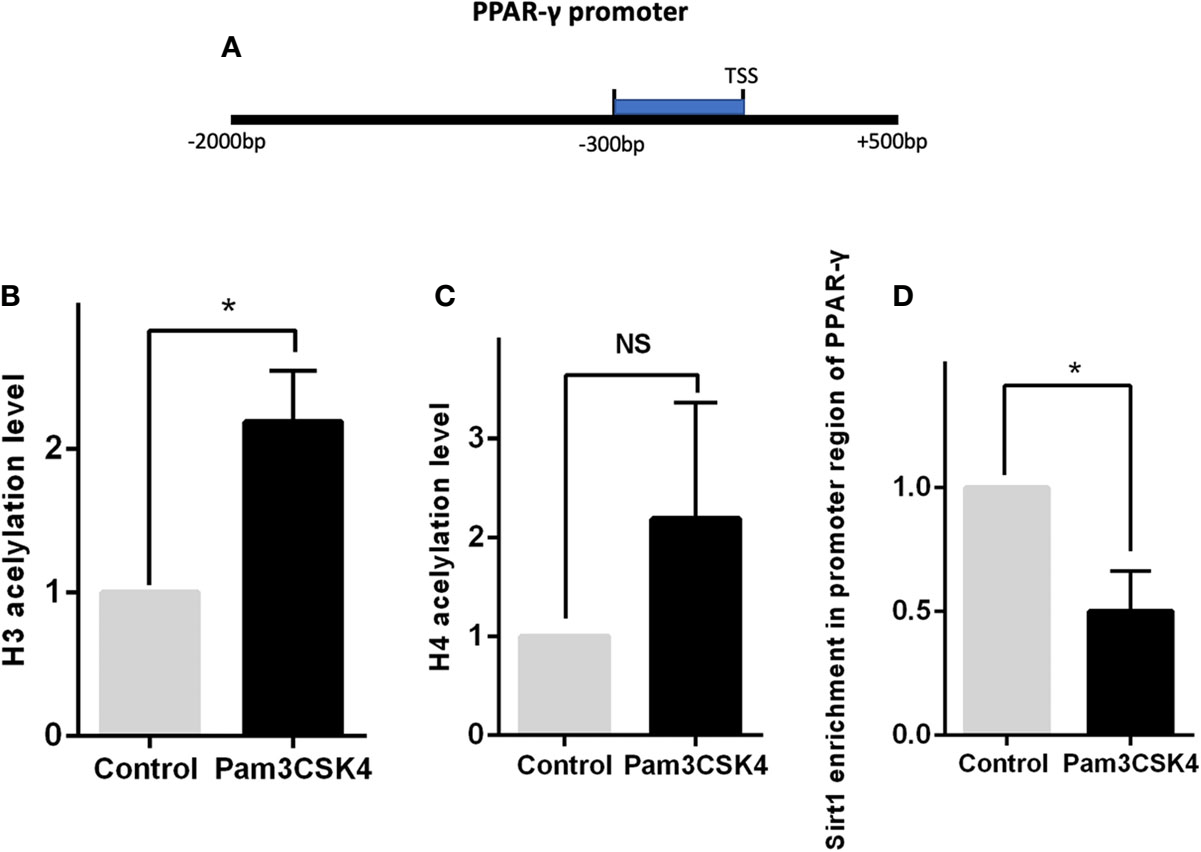
Histone deacetylase Sirt1 modulates PPAR-γ expression through histone modification. **(A)** Diagram depicting the studied region of PPAR-γ promoter sequences. **(B)** The ChIP-qPCR results showed that the H3 acetylation level in the promoter region of PPAR-γ increased significantly by Pam3CSK4 compared with the control group. **(C)** The H4 acetylation level showed no significant changes between the two groups. **(D)** The enrichment level of Sirt1 in the promoter region of PPAR-γ was significantly decreased in the Pam3CSK4 group detected by ChIP-qPCR. Data represent the mean of three independent experiments (*P < 0.05). NS, No statistical significance.

Previous studies have demonstrated that expression of PPAR-γ is regulated by multiple epigenetic modifications. To explore the detailed mechanism by which Pam3CSK4 pretreatment induces PPAR-γ expression, we conducted another *in vitro* experiment. First, we collected the CD14^+^ monocytes after stimulation with or without Pam3CSK4 for 48 h and detected the histone(H)3 and H4 acetylation levels in the PPAR-γ promoter by ChIP-qPCR. We used the JASPAR database (http://jaspar.genereg.net/) to predict the TFs binding sites of PPAR-γ promoter region and we found that the region from −300- to 0–base pair (bp) upstream of transcription starting site (TSS) is a high-density area of TFs and a key modulatory region. Therefore, we evaluated the histone acetylation levels of H3 and H4 and the enrichment of Silent information regulator 1 (Sirt1) in this region (**Figure 7A**). The nucleotide sequence of this region is provided in **Supplementary Data Sheet 1**. We observed that the H3 acetylation level significantly elevated in the Pam3CSK4-treated group compared with the control group, while the H4 acetylation level exhibited no significant changes (**Figures 7B, C**). The histone acetylation state is determined by histone acetyltransferases (HATs), such as P300/CBP, and histone deacetylases (HDACs), such as HDAC1 and Sirt1. Therefore, we determined whether the H3 acetylation level in the PPAR-γ promoter was regulated by histone modification enzymes. The chromatin immunoprecipitation (ChIP)–qPCR assays showed that the enrichment level of Sirt1 in the PPAR-γ promoter was significantly decreased in the Pam3CSK4 group (**Figure 7D**). These data indicated that Pam3CSK4 pretreatment modulates PPAR-γ expression by regulating histone modification through the inhibition of Sirt1 binding to the PPAR-γ promoter.

## Discussion

Compelling evidence suggests pivotal roles for monocytes and monocyte-derived macrophages (MDMs) in autoimmune diseases, including SLE (23, 24). Monocytes are an essential part of innate immunity. It is traditionally believed that defects in the phagocytosis and antigen presentation functions of monocytes result in the accumulation of apoptotic debris, leading to autoimmune responses (11, 25). The excessive secretion of pro-inflammatory cytokines from circulating and tissue-infiltrating Mo/Mϕ facilitates perpetuated inflammation in SLE patients (26). Recent studies have increasingly focused on aberrations of monocytes and MDMs in SLE. M1-like macrophages derived from monocytes can defend the host against infection. However, these M1-like macrophages exhibit pro-inflammatory properties in SLE patients, whereas the reduced populations of M2-like macrophages derived from monocytes play an immunomodulatory role (27, 28). The circulating monocytes from SLE patients displayed an M1-like phenotype that was influenced by the environment, including the presence of cytokines and the elevated level of high mobility group protein-1 (HMGB1) (12). Macrophage polarization toward the M1 phenotype is associated with flare in SLE patients (15). Murine studies showed that the adoptive transfer of M2 macrophages significantly reduced SLE severity in lupus-prone mice (16). Moreover, macrophages with transmembrane activator and calcium-modulator and cyclophilin interactor (TACI)–deficient present an M2-skewed phenotype and improved the survival of MRL-Fas/Lpr mice, and the transfer of macrophages from TACI−/− Lpr-mice ameliorated SLE-like symptoms in age-matched, sick, wild-type mice (29).

Some drugs can induce the differentiation of monocytes into an M2-like phenotype and alleviate SLE symptoms. Indole-3-carbinol (I3C) is a ligand of the aryl hydrocarbon receptor. Upon ligation, I3C could regulate inflammatory responses and cytokine expression in murine models of SLE and prolong the lifespan of lupus-prone mice (30, 31). *Ex vivo* experiments demonstrated the effects of I3C on the skewing of MDMs from SLE patients toward the M2-like phenotype, which inhibited the secretion of pro-inflammatory cytokines and enhance the secretion of anti-inflammatory cytokines (32). Sodium valproate (VPA), as a histone deacetylase inhibitor, could successfully promote the anti-inflammatory reaction by supporting the polarization of MDMs from SLE patients toward the M2-like phenotype (33). Another study also reported that Pam3CSK4, a TLR2 agonist, could stimulate SLE patient-derived monocytes to differentiate into M2-like macrophages, leading to an improved outcome of murine lupus (34). Moreover, pioglitazone, a PPAR-γ agonist, could induce the generation of the M2 population and consequently enhance immunomodulation in SLE patients (21).

Our results also demonstrated that Pam3CSK4 could drive MDMs differentiate into an M2-like subset with increased Arg-1 expression and decreased expression of CD80, NF-κB, and pro-inflammatory cytokines IL-1b, IL-6, and IL-12, as well as CCR7. Arg-1 is a surrogate for polarized alternative macrophages and is significant enhancement to the production of nitric oxide (35). Increased Arg-1 along with decreased CD80 are regarded as markers of the M2-like phenotype of MDMs (36). IL-1b, IL-6, and IL-12 are associated with the M1-like phenotype and are elevated in a multitude of immune-mediated diseases, including SLE (36–39). CCR7 is a chemokine receptor that interacts with CCL19 and CCL21. Increased expression of CCR7 supports migration to lymph nodes and potentially drives the inappropriate localization of autoantigen-presenting cells (40). Pam3CSK4 decreased the expression of CCR7, which may impede the migration of MDMs. However, further exploration of the role of downregulated CCR7 expression on monocytes in the pathogenesis of SLE is needed. NF-κB acts as a TF that promotes the transcription of many pro-inflammatory cytokines (4). The decreased expression of IL-1b, IL-6, and IL-12 may be attributed to the downregulation of NF-κB by Pam3CSK4.

PPAR-γ is a nuclear hormone receptor that acts as a ligand-activated transcriptional factor (41). PPAR-γ can inhibit the activation and regulate the differentiation of macrophages and consequently ameliorate SLE (42). As mentioned above, the PPAR-γ agonist pioglitazone could enhance the alternative activation of MDMs from SLE patients (21). Furthermore, mouse experiments revealed that mice lacking PPAR-γ expression in macrophages showed deficiencies in phagocytosis and developed autoimmune glomerulonephritis (43). And the expression and activity of PPAR-γ decreased in MRL/Lpr-derived primary mesangial cells which further exacerbated the disease state (44). However, rosiglitazone, a PPAR-γ agonist, decreased renal injury in a female mouse model of SLE (45). To investigate the mechanism by which Pam3CSK4 regulates MDM polarization, we measured the expression level of PPAR-γ and found that PPAR-γ expression increased over time. Inhibiting PPAR-γ with its antagonist T0070709 reversed the modulatory effects of Pam3CSK4 on MDMs. These results demonstrated that Pam3CSK4 regulates the polarization of MDMs via the activation of PPAR-γ. In addition, we verified that the mRNA and protein levels of PPAR-γ were increased in monocytes from SLE patients, specifically in patients with anti-dsDNA antibodies, which suggested that PPAR-γ may act as a feedback regulator that ameliorates SLE.

To further understand how Pam3CSK4 upregulated PPAR-γ expression, we detected the histone H3 and H4 acetylation levels in the PPAR-γ promoter. We found that the histone H3, but not H4, acetylation levels significantly increased in the promoter region of PPAR-γ upon Pam3CSK4 stimulation. Moreover, the enrichment of the deacetylase Sirt1 significantly decreased in the same region of PPAR-γ promoter, which indicated that Pam3SCK4 could decrease the enrichment of Sirt1, thus increasing the histone acetylation level and promoting the expression level of PPAR-γ. Histone acetylation is a kind of epigenetic modification (46). Histone acetylation favors gene transcription due to the relatively uncondensed chromatin status (47). Sirt1 is an NAD-dependent lysine deacetylase that can modulate the histone acetylation status (48). The relationship between Sirt1 and TLR2 activation was observed in a previous study, which proved that TLR2 ligation was associated with decreased Sirt1 expression (49). Our results particularly highlight the relationship between TLR2 ligation and Sirt1 enrichment in the promoter region, and these results are more convincing. Interestingly, the roles of Sirt1 in SLE are different in different cell types. Expression of Sirt1 increased significantly in CD4^+^T cells from patients with active lupus which indicated increased Sirt1 expression is related to the development of SLE (50). However, another study pointed out that ultraviolet B (UVB) exposure could inhibit activity of DNA methyltransferase1 (DNMT1) via suppression of Sirt1 in CD4^+^T cells from SLE patients which implied that inhibited expression of Sirt1 contributed to the pathogenesis of SLE (51). Recently, Eri Katsuyama et al. found that decreased expression of Sirt1 in CD8CD38^high^ T cells participated in the mediation of cytotoxicity of CD8 T cells and was associated with the infection tendency in SLE patients (52). These results showed that Sirt1 exhibited antithetic effects under different circumstances.

## Conclusion

Our results demonstrate that Pam3CSK4 can induce monocytes to polarize toward an M2-like phenotype with increased expression of Arg-1 and decreased expression of CD80, NF-κB, IL-1b, IL-6, IL-12, and CCR7. This process is mediated by PPAR-γ, whose expression is elevated in the monocytes from SLE patients. Pam3CSK4 regulates PPAR-γ expression by downregulating Sirt1 enrichment in the PPAR-γ promoter and consequently increasing H3 acetylation. Taken together, our results suggest a protective role of the TLR2/Sirt1/PPAR-γ signaling pathway in the pathogenesis of SLE and shed light on potentially novel treatment strategies.

## Standard Biosecurity and Institutional Safety Procedures

All the biosafety measurements have been adopted and the institutional safety procedures are adhered. The laboratory of our institution has biosafety level 1 (BSL-1) standard where all standards and protocols are adopted as per the guidelines of CLSI.

## Data Availability Statement

The raw data supporting the conclusions of this article will be made available by the authors, without undue reservation.

## Ethics Statement

The studies involving human participants were reviewed and approved by The Human Ethics Committee of the Second Xiangya Hospital. The patients/participants provided their written informed consent to participate in this study.

## Authors Contributions

YLiu performed most of the experiments, analyzed the data, and wrote the manuscript. SL and YLi collected the clinical samples and evaluated the SLEDAI scores of SLE patients. YZ, JW, and RZ critically revised the manuscript and provided technical support and suggestions. QL and JZ designed and supervised the study. All authors contributed to the article and approved the submitted version.

## Funding

This work was supported by grants from the National Natural Science Foundation of China (No. 81502732, No. 81974477, No. 81830097, and No. 82073448) and the innovation project of the Chinese Academy of Medical Sciences (Research Unit, No. 2019-I2M-5–033).

## Conflict of Interest

The authors declare that the research was conducted in the absence of any commercial or financial relationships that could be construed as a potential conflict of interest.

## Abbreviations

ARG1, Arginase-1; CD, cluster of differentiation; ChIP, Chromatin Immunoprecipitation; CCL, chemokines; CCR, C-C chemokine receptor; DC, dendritic cell; H3(4), Histone 3(4); HATs, histone acetyltransferases; HDACs, histone deacetylases; HLA, Human leukocyte antigen; HMGB1, High mobility group protein-1; I3C, Indole-3-carbinol; IL, Interleukin; MDMs, monocyte-derived macrophages; NF-κB, nuclear transcription factor-κB; PBMC, peripheral blood mononuclear cell; PBS, Phosphate Buffered Saline; PCR, Polymerase Chain Reaction; PPAR, Peroxisome proliferator activated receptor; Sirt1, Silent information regulator 1; SLE, Systemic lupus erythematosus; TACI, transmembrane activator and calcium-modulator and cyclophilin interactor; TLR, Toll-like receptor; VPA, Sodium valproate.
