# Supplementary material for: Increased Expression of PPAR-γ Modulates Monocytes Into a M2-Like Phenotype in SLE Patients: An Implicative Protective Mechanism and Potential Therapeutic Strategy of Systemic Lupus Erythematosus
Source: Front Immunol. 2021 Jan 19;11:579372. doi: 10.3389/fimmu.2020.579372 (PMC7873911; doi:10.3389/fimmu.2020.579372)
Supplement: Supplementary file 1 [file DataSheet_1.pdf]

1700-2000（-300bp~0bp）

>NC_000003.12:12287550-12287850 Homo sapiens chromosome 3, GRCh38.p7 Primary Assembly

CACACCTCGGTCTCCCCAGACCGGCCCTGGCCGGGGGCATCCCCCTAAACTTCGGATCCCTCCTCGGAA
ATGGGACCCTCTCTGGGCCGCCTCCCAGCGGTGGTGGCGAGGAGCAAACGACACCAGGTAGCCTGCCGC
GGGGCAGAGAGTGGACGCGGGAAAGCCGGTGGCTCCCGCCGTGGGCCCTACTGTGCGCGGGCGGCGGCC
GAGCCCGGGCCGCTCCCTCCCAGTCGCGCGCCGCCGCCCCCGCCCCCGCCCCCGCCCCCGCCCCCACCC
CCACCCCCACCCCCACCCCCAGCCG
