# Supplementary material for: Increased Expression of PPAR-γ Modulates Monocytes Into a M2-Like Phenotype in SLE Patients: An Implicative Protective Mechanism and Potential Therapeutic Strategy of Systemic Lupus Erythematosus
Source: Front Immunol. 2021 Jan 19;11:579372. doi: 10.3389/fimmu.2020.579372 (PMC7873911; doi:10.3389/fimmu.2020.579372)
Supplement: Supplementary file 3 [file Table_2.pdf]

Supplementary Table 2. RT-PCR primer sequences.

H-CCR7-F: 5′-ATTACTACAACCGATCCACCTC-3′	H-CCR7-R: 5′-TAGGAGCATGCCACTGAAGA-3′
H-CD80-F: 5′-CCATTGTGATCCTGGCTCTG-3′	H-CD80-R: 5′-CGTCACTTCAGCCAGGTGTT-3′
H-IL-1β-F: 5′- GTACCTGAGCTCGCCAGTGA-3′	H-IL-1β-R: 5′-TGGTGGTCGGAGATTCGTAG-3′
H-IL-12-F: 5′- TCCTCCTGGACCACCTCAGT-3′	H-IL-12-R: 5′-CTGGCCTTCTGGAGCATGTT-3′
H-ARG1-F: 5′- TCATCTGGGTGGATGCTCACAC-3′	H-ARG1-R: 5′- GAGAATCCTGGCACATCGGGAA-3′
H-PPAR-γ-F: 5’- CCGCAGATTTGAAAGAAG-3’	H-PPAR-γ-R: 5’-AAGGAGTGGGAGTGGTCT-3’
H-P50-F: 5′-TCTGCTTCCAGGTGACAGTG-3′	H-P50-R: 5′-ATCTTGAGCTCGGCAGTGTT-3′
H-GAPDH-F: 5′-GACAAGCTTCCCGTTCTCAG-3′	H-GAPDH-R: 5′-GAGTCAACGGATTTGGTCGT-3′

Note: H = human; F = forward primer; and R = reverse primer.
