# Supplementary material for: Increased Expression of PPAR-γ Modulates Monocytes Into a M2-Like Phenotype in SLE Patients: An Implicative Protective Mechanism and Potential Therapeutic Strategy of Systemic Lupus Erythematosus
Source: Front Immunol. 2021 Jan 19;11:579372. doi: 10.3389/fimmu.2020.579372 (PMC7873911; doi:10.3389/fimmu.2020.579372)
Supplement: Supplementary file 4 [file Table_3.pdf]

Supplementary Table 3. ChIP-qPCR primer sequences.

H-PPAR-γ-F: 5’- GGGGCATCCCCCTAAACTTC-3’	H-PPAR-γ-R: 5’-AGGCTACCTGGTGTCGTTTG-3’

Note: H = human; F = forward primer; and R = reverse primer.
